# Ginsenoside Rc from Panax Ginseng Ameliorates Palmitate-Induced UB/OC-2 Cochlear Cell Injury

**DOI:** 10.3390/ijms24087345

**Published:** 2023-04-16

**Authors:** Nicholas B. Gill, Presley D. Dowker-Key, Katelin Hubbard, Brynn H. Voy, Jay Whelan, Mark Hedrick, Ahmed Bettaieb

**Affiliations:** 1 Department of Nutrition, University of Tennessee Knoxville, Knoxville, TN 37996-1920, USA; 2 Department of Animal Science, University of Tennessee Institute of Agriculture, Knoxville, TN 37996-0840, USA; 3 Graduate School of Genome Science and Technology, University of Tennessee, Knoxville, TN 37996-0840, USA; 4 Department of Audiology and Speech Pathology, The University of Tennessee Health Science Center, Knoxville, TN 37996-0240, USA; 5 Department of Biochemistry, Cellular and Molecular Biology, University of Tennessee, Knoxville, TN 37996-0840, USA

**Keywords:** cochlear hair cells, ginseng, inflammation, oxidative stress, ER stress, apoptosis, phytochemical, hearing

## Abstract

By 2050, at least 700 million people will require hearing therapy while 2.5 billion are projected to suffer from hearing loss. Sensorineural hearing loss (SNHL) arises from the inability of the inner ear to convert fluid waves into neural electric signals because of injury to cochlear hair cells that has resulted in their death. In addition, systemic chronic inflammation implicated in other pathologies may exacerbate cell death leading to SNHL. Phytochemicals have emerged as a possible solution because of the growing evidence of their anti-inflammatory, antioxidant, and anti-apoptotic properties. Ginseng and its bioactive molecules, ginsenosides, exhibit effects that suppress pro-inflammatory signaling and protect against apoptosis. In the current study, we investigated the effects of ginsenoside Rc (G-Rc) on UB/OC-2 primary murine sensory hair cell survival in response to palmitate-induced injury. G-Rc promoted UB/OC-2 cell survival and cell cycle progression. Additionally, G-Rc enhanced the differentiation of UB/OC-2 cells into functional sensory hair cells and alleviated palmitate-induced inflammation, endoplasmic reticulum stress, and apoptosis. The current study offers novel insights into the effects of G-Rc as a potential adjuvant for SNHL and warrants further studies elucidating the molecular mechanisms.

## 1. Introduction

It is estimated that 30 million Americans suffer from hearing loss, a condition that can affect all groups despite age, race/ethnicity, or socioeconomic status [1]. Unfortunately, many more individuals may endure some extent of hearing loss, but their condition goes untreated due to underdiagnosis by physicians or low accessibility to healthcare. An estimated two-thirds of the population over 70 years old suffers from hearing loss; therefore, like type 2 diabetes (T2D), the risk of hearing loss greatly increases later in life [2]. Hearing loss can be classified into three groups: sensorineural, conductive, and mixed. Alterations to the ability of the inner ear to convert fluid waves into neural electric signals may lead to sensorineural hearing loss (SNHL). Age-related hearing loss, also known as presbycusis, is the most prevalent type of SNHL and can be attributed to different factors, such as genetics, oxidative stress, inflammation, cochlear vascular changes, noise exposure, and ototoxic drugs [1,3]. Conductive hearing loss is characterized by the dysfunction of the outer and middle ear machinery to transduce sound into mechanical energy [4]. Pathologies leading to conductive hearing loss generally manifest as morphological changes in the structures of the outer and middle ear. Lastly, mixed hearing loss presents both conductive and sensorineural components.

SNHL, resulting from an injury to the cochlear and supporting cells, such as noise exposure, is attributed to underlying pathophysiological mechanisms. Injury to the inner ear provokes an immune response in the cochlear hair cells characterized by the presence of macrophages and pro-inflammatory cytokines [5]. Although cochlear hair cells endogenously express markers of inflammation, M1 macrophages respond as an acute response to injury and stimulate the production of pro-inflammatory cytokines via secreting ligands that bind multiple types of pattern-recognition receptors [5]. The increase in inflammatory signaling can scar the inner ear structures and result in a reduction of auditory signal transduction. Aging and chronic inflammation are highly associated with hearing loss; therefore, chronic inflammation of the inner ear may occur concomitantly with the normal aging process. A proposed cause includes the altered ability of hearing machinery to regulate acute immune responses in older tissues, thus leading to an increase of pro-inflammatory markers over time [6]. Furthermore, chronic inflammation is observed in pathologies such as cardiovascular disease (CVD), T2D, and Alzheimer’s disease, all of which are correlated with SNHL [6]. From these associations, it can be deduced that hearing loss may manifest from chronic inflammation and could be exacerbated by other disease states.

Emerging evidence indicates that phytochemicals exhibit antioxidative, anti-inflammatory, and neuroprotective properties. Phytochemicals refer to a broad class of bioactive molecules derived from plants that have robust effects on cell signaling. Additionally, many phytochemicals were demonstrated to synergistically regulate a plethora of biochemical and physiological processes [7,8,9]. Currently, molecules such as isoflavones from various species, catechins from green tea, and capsaicin from peppers have been shown to possess inhibitory effects on tumorigenesis and are being further investigated as possible adjuvants to pharmaceuticals and chemotherapy [10]. Phenols, a large class of bioactive molecules, are important for the modulation of T2D and insulin resistance due to their ability to reduce pro-inflammatory cytokines, such as interleukin-1β (IL-1β), interleukin-6 (IL-6), and tumor necrosis factor-α (TNF-α). Moreover, phytochemicals can decrease the DNA-binding efficiency of nuclear factor kappa B (NF-κB), thus reducing the expression of certain inflammatory markers [11]. 

Ginseng, a traditional Chinese medicinal plant, is now being investigated to elucidate its useful properties. Ginseng is a slow-growing plant related to various types of ivy. Although multiple species of ginseng grow natively worldwide, its medicinal use originates from the East, particularly China. Ginseng native to the Asian region has been a long-standing constituent of traditional Chinese medicine (TCM), with the first documentation of its use being 2000 years ago. *Panax ginseng,* also known as Asian or Korean ginseng is commonly used for several medicinal purposes. *For instance, Panax ginseng* is frequently used in TCM to treat anorexia, impotence, palpitations, insomnia, and shortness of breath [12]. 

Ginseng exerts its effects across multiple pathways of the nervous system and immune system, and in energy metabolism [13]. It has been proposed that the anti-inflammatory effects of ginseng are dependent on the combined actions of the bioactive molecules within ginseng, known as ginsenosides, as well as their metabolized product, compound K (CK). Each ginsenoside has its isolated effects, but together they potently downregulate pro-inflammatory cytokines and increase anti-inflammatory cytokines to reduce inflammation [14]. For example, ginsenoside Rb1 (G-Rb1) has been shown to inhibit TNF-α production and reduce activation of the inflammatory regulator, NF-κB [15]. Moreover, CK and ginsenoside Rc (G-Rc) have been also shown to reduce the generation of reactive oxygen species (ROS) through the suppression of superoxide-induced free radicals [16,17]. G-Rc is one of the most prevalent ginsenosides in *P. ginseng*. In addition to its role in reducing oxidative stress, G-Rc is shown to reduce TNF-α, IL-1β, and the activation of TANK-binding kinase-1 (TBK1)/interferon regulatory factor-3 (IRF-3) and p38/activating transcription factor-2 (ATF-2), both well-established pro-inflammatory signaling pathways [18]. However, despite ginseng’s role in reducing inflammation related to a variety of pathologies, ginseng’s potential to alleviate hearing loss has yet to be fully established. Fujita and colleagues demonstrated that intravenous infusion of G-Rb1 to gerbils protected against damage to the spiral ganglion cells after cochlear ischemia [19]. In line with these findings, Durankaya and colleagues demonstrated that the administration of Korean red ginseng (KRG) to Wistar albino rats reduced noise-induced apoptotic cell death in the organ of Corti and spiral ganglion and, furthermore, provided protection from hearing loss [20]. Likewise, *P. ginseng* was demonstrated to reduce noise-induced temporary threshold shifts in textile workers after 14 days of treatment and to improve symptoms of tinnitus in a group of chronic tinnitus patients [21,22]. 

Despite the evident beneficial effects of various ginsenosides in protecting against stress-induced hearing loss, the molecular mechanisms remain elusive. Importantly, additional mechanistic studies are needed to better describe the role of ginsenosides in preserving hearing machinery. In the current study, we focused our efforts on investigating the effects of G-Rc on cell survival and homeostasis in response to palmitate-induced inflammation and cochlear cell injury.

## 2. Results

### 2.1. UB/OC-2 Cells Differentiate into Functional Cochlear Hair Cells

**Figure 1:**
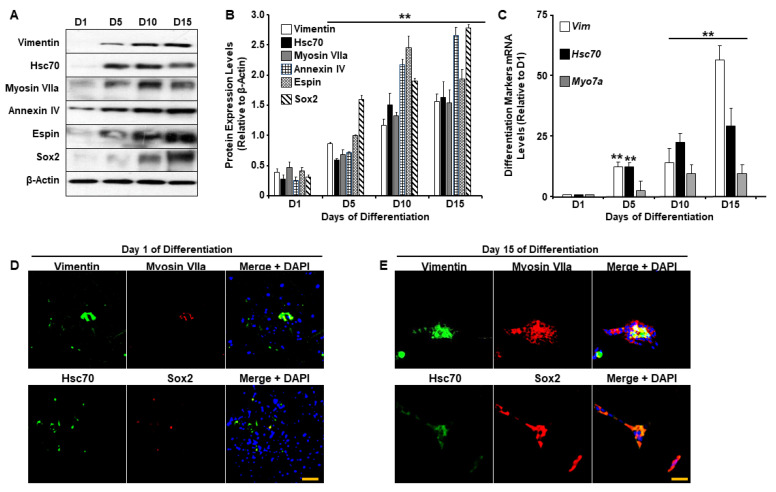
UB/OC-2 cells differentiate into sensory hair cells. (**A**,**B**) Representative immunoblots (**A**) and bar graph quantitative assessment (**B**) of Vimentin, Hsc70, Myosin VIIa, Espin, Annexin IV, and Sox2 in total cell lysates from UB/OC-2 during differentiation. Data is representative of at least three independent experiments. (**C**) mRNA levels of differentiation markers *Vim*, *Hsc70*, and *Myo7a*. Data are normalized to *Tbp* and are representative of at least three independent experiments. In (**B**,**C**), ** *p* < 0.01 indicates a significant difference between cells on the indicated day of differentiation (D5, D10, or D15) and undifferentiated cells (D1). (**D**,**E**) Immunofluorescence of Vimentin, Hsc70 (green fluorescence), Sox2, Myosin VIIa (red fluorescence), and nuclear DNA using DAPI (blue fluorescence) in undifferentiated (Day 1; (**D**)) and differentiated (Day 15; (**E**)) UB/OC-2 cells. Scale bar = 100 μm.

It is well-established that sensory hair cells in the mammalian inner ear are exceptionally sensitive to various genetic and environmental stressors and lack spontaneous regenerative capacity. As such, injuries leading to cochlear hair cell death often result in permanent hearing loss [23,24]. Experimental attempts that aim to prevent and treat insults to hair cells have been hampered by the limited ability to use hair cells in a laboratory setting and are further impeded by a lack of understanding of how these cells behave and function in vitro. Recent studies have demonstrated that UB/OC-2 primary murine cells could differentiate into cochlear hair cells. This process of differentiation is accompanied by the expression of several specific markers including Myosin VIIa, Parvalbumin 3, Purkinje cell protein 4 (PCP4), Annexin IV, Espin, and SRY-box 2 (Sox2) [25,26], among others. Therefore, before investigating the effects of G-Rc on hair cell homeostasis, we first confirmed the differentiation potential of UB/OC-2 cells by examining changes in the expression of Vimentin, Heat shock cognate 70 (Hsc70), Myosin VIIa, Annexin IV, Espin, and Sox2 upon differentiation. As shown in Figure 1A,B, immunoblotting revealed an increase in the expression of all proteins upon incubation of the cells for 15 days at 38 °C. The levels of these proteins were significantly higher after 5, 10, and 15 days of differentiation (Figure 1B). Consistent with these findings, *Vim*, *Hsc70*, and *Myo7a* mRNA levels (Figure 1C) were also significantly higher on days 10 and 15 of differentiation. To further validate our experimental model, we assessed the expression of Vimentin, Myosin VIIa, Hsc70, and Sox2 by immunofluorescence. As demonstrated in Figure 1D,E, on day 15 of differentiation (Figure 1E), UB/OC-2 cells expressed higher levels of Vimentin, Myosin VIIa, Hsc70, and Sox2 compared to undifferentiated cells (Figure 1D). Together these data confirm and validate previous studies on the ability of UB/OC-2 cells to differentiate into functional cochlear hair cells [27,28].

### 2.2. Effects of G-Rc on Cell Survival/Toxicity, Proliferation, and Cell Cycle

**Figure 2:**
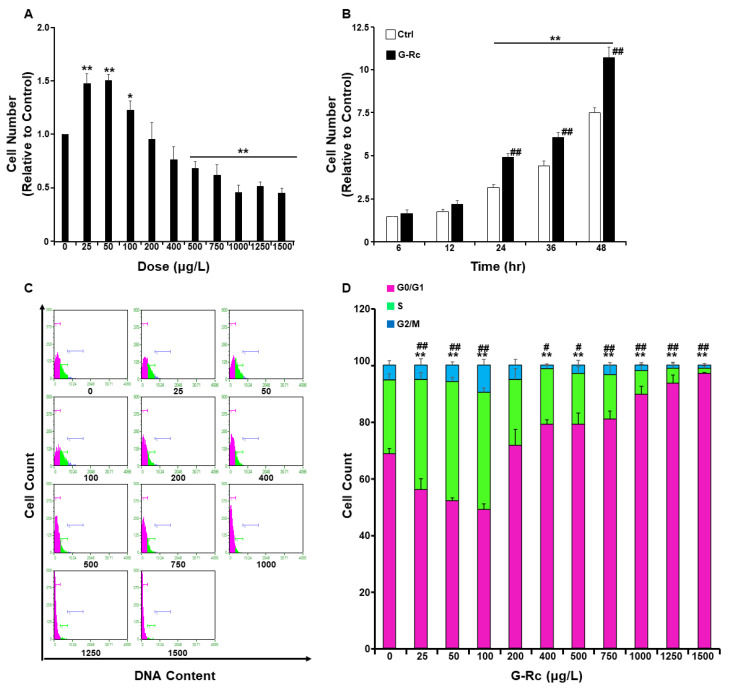
Effects of G-Rc on cell survival/toxicity, proliferation, and cell cycle. (**A**) Effects of G-Rc on cell survival using the MTT method. Undifferentiated UB/OC-2 cells were treated with increasing doses of G-Rc for 24 h. Bar graphs represent the intensity of formazan (produced from MTT by viable cells) staining reflective of the cell number and presented as means + SEM. (**B**) Effects of G-Rc on cell proliferation using the SRB method: cells were treated with G-Rc (25 μg/L) for up to 48 h. Bar graphs represent the intensity of SRB staining reflective of the cell number and presented as means + SEM. (**C**) Cell cycle analysis and assessment of DNA content in undifferentiated UB/OC-2 cells treated with the indicated concentration (0–1500 μg/L) of G-Rc for 24 h. A representative histogram for each treatment is shown. (**D**) Bar graphs represent the percentages of cells in each phase of the cell cycle, which were estimated using the GuavaSuite Software package, and are presented as means + SEM from three independent experiments. In (**A**), * *p* < 0.05 and ** *p* < 0.01 indicate a significant difference between the indicated concentration of G-Rc and control cells non-treated with G-Rc. In (**B**), ** *p* < 0.01 indicates a significant difference between the indicated time and control cells at 6 h post-seeding. ^##^ *p* < 0.01 indicates a significant difference between G-Rc-treated cells and control cells non-treated with G-Rc. In (**D**), ** *p* < 0.01 indicates a significant difference in DNA content between G-Rc-treated cells and control cells non-treated with G-Rc while at the G0/G1 phase. ^#^ *p* < 0.05 and ^##^ *p* < 0.01 indicate a significant difference in DNA content between G-Rc-treated cells and control cells non-treated with G-Rc while at S phases.

Recent studies have highlighted the potential of ginsenosides to protect against hearing loss [19,20]. However, the effects of ginsenosides, G-Rc in particular, on cellular homeostasis and on the response to pro-inflammatory stimuli remain to be determined. Thus, we first examined the effects of varying doses of G-Rc on the survival and proliferation of undifferentiated hair cells. At low doses (≤100 μg/L), G-Rc caused a significant increase in UB/OC-2 cell proliferation (Figure 2A). However, upon treatment of UB/OC-2 cells with higher concentrations of G-Rc (≥500 μg/L), a significant decrease in cell number was observed (Figure 2A).

A recent study has reported that the concentration of G-Rc in circulation upon repeated consumption by 15 healthy subjects for 15 days reached a maximum of 9.69 ± 7.08 μg/L [29]. Therefore, we investigated the effects of G-Rc on cell proliferation at a relatively physiologically relevant dose of G-Rc (25 μg/L). As shown in Figure 2B, treatment of UB/OC-2 cells with G-Rc at 25 μg/L significantly increased cell proliferation at 24, 36, and 48 h compared to untreated control cells. In follow-up experiments, cell cycle analysis demonstrated that, at physiologically relevant doses (25 µg/L), G-Rc caused a significant increase in cellular DNA content (Figure 2C,D). Consistent with the cell toxicity data, treatment of UB/OC-2 cells with higher concentrations of G-Rc (≥500 μg/L) resulted in a reduction in cell cycle progression through the G0/G1 phase (Figure 2C,D). Taken together, these results suggest that G-Rc-induced toxicity and inhibition of cell proliferation at high doses is mediated, at least in part, through cell cycle arrest in the G0/G1 phase. However, at low concentrations, G-Rc promoted cell survival and cycle cycle progression. Hence, we used a human physiologically relevant fixed dose of G-Rc (25 µg/L) in follow-up experiments.

### 2.3. A Physiologically Relevant Dose of G-Rc Promotes the Differentiation of UB/OC-2 Cells into Cochlear Hair Cells

**Figure 3:**
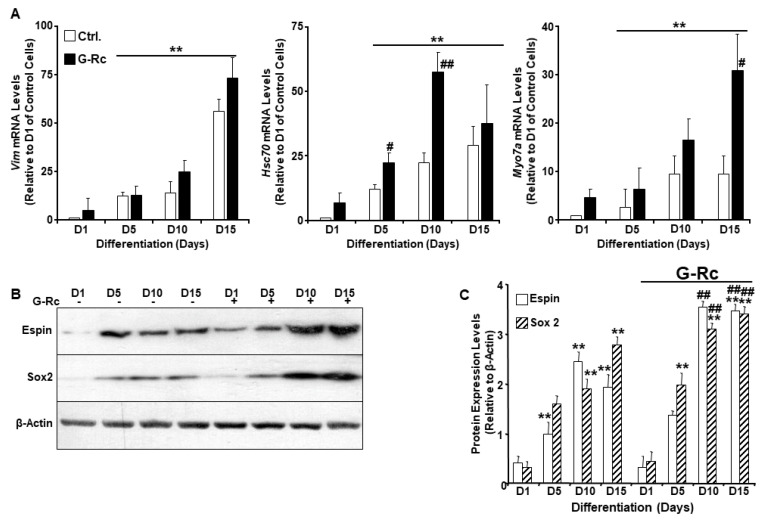
A human physiologically relevant dose of G-Rc promotes the differentiation of UB/OC-2 into functional sensory hair cells. (**A**) Quantitative (q)RT-PCR of *Vim*, *Hsc70*, and *Myo7a* mRNA levels in control and G-Rc (25 μg/L) treated cells on days 1, 5, 10, and 15 of differentiation. Data are normalized to *Tbp* and expressed as fold change relative to undifferentiated (D1) control cells non-treated with G-Rc. Results are representative of three independent experiments and data are expressed as mean + SEM. ** *p* < 0.01 denotes a significant difference between indicated time points and D1 for each treatment. ^#^ *p* < 0.05 and ^##^ *p* < 0.01 indicate significant difference between G-Rc-treated and control cells non-treated with G-Rc. (**B**,**C**) Representative immunoblots (**B**) and bar graph quantitative assessment (**C**) of Espin and Sox2 in total cell lysates from differentiating UB/OC-2 with and without G-Rc treatment(25 μg/L). Results are representative of three independent experiments, and data are expressed as mean + SEM. ** *p* < 0.01 indicates a significant difference between indicated time points and D1 for each treatment. ^##^ *p* < 0.01 indicates significant difference between G-Rc-treated and control cells non-treated with G-Rc.

Ginsenosides exhibit protective effects against ischemia-induced damage to the spiral ganglion cells and reduce noise-induced apoptotic cell death in the organ of Corti and spiral ganglion [20]. However, the effects of G-Rc on cochlear hair cell differentiation remain elusive. We, therefore, investigated the effects of G-Rc on UB/OC-2 differentiation by evaluating the expression of *Vim*, *Hsc70*, and *Myo7a* upon differentiation using quantitative real-time polymerase chain reaction (qRT-PCR). While G-Rc had no effects on *Vim* expression, a significant increase in *Hsc70* on days 5 and 10 of differentiation was observed in cells treated with G-Rc throughout the differentiation process (Figure 3A). On the other hand, a significant increase in *Myo7a* levels was only observed on day 15 of differentiation (Figure 3A). To support the effects of G-Rc on UB/OC-2 cell differentiation, the expression of Espin and Sox2 was also evaluated by Western blot. Treatment of UB/OC-2 cells with G-Rc enhanced the expression of differentiation markers Espin and Sox2 throughout the differentiation process (Figure 3B,C). These data suggest a positive effect of G-Rc on cochlear hair cell differentiation. However, a thorough characterization of G-Rc’s effects on cochlear hair cell function is warranted.

### 2.4. A Physiologically Relevant Dose of G-Rc Alleviates Palmitate-Induced Alterations to Cell Survival and Proliferation, Oxidative Stress, and Inflammation

**Figure 4:**
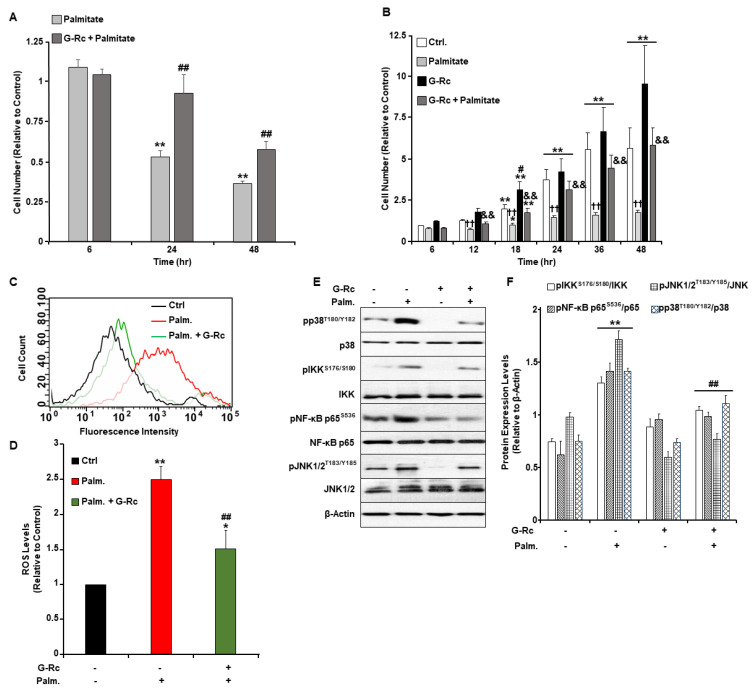
A physiologically relevant dose of G-Rc alleviates palmitate-induced alterations to cell survival and proliferation, oxidative stress, and inflammation. (**A**) Cell toxicity assay using the MTT method in differentiated UB/OC-2 cells treated with a freshly prepared solution of palmitate alone (0.5 mM) or in combination with G-Rc (25 μg/L) for up to 48 h. Bar graphs represent the intensity of formazan staining reflective of the cell number and are presented as means + SEM. ** *p* < 0.01 indicates a significant difference between palmitate and non-palmitate-treated cells. ^##^ *p* < 0.01 indicates a significant difference between palmitate/G-Rc-treated cells and cells treated with palmitate alone. (**B**) Effects of G-Rc on palmitate-induced cell proliferation using the SRB method. Cells were treated with palmitate alone, (0.5 mM), G-Rc (25 μg/L) alone, or a combination of palmitate and G-Rc for up to 48 h. Bar graphs represent the intensity of SRB staining reflective of the cell number and are presented as means + SEM. * *p* < 0.05 and ** *p* < 0.01 indicate a significant difference between the indicated time and control cells at 6 h post-seeding. ^#^ *p* < 0.05 indicates a significant difference between G-Rc-treated cells (non-palmitate-treated) and control cells non-treated with G-Rc nor palmitate. †† *p* < 0.01 indicates a significant difference between cells treated with palmitate alone versus control cells non-treated with palmitate nor G-Rc. && *p* < 0.01 indicates a significant difference between cells treated with a combination of palmitate and G-Rc versus cells treated with palmitate alone. (**C**,**D**) Flow cytometry assessment of ROS production as determined using CM- DCH_2_F-DA in fully differentiated UB/OC-2 cells treated with palmitate or palmitate in combination with G-Rc (25 μg/L). A representative histogram for each treatment from three independent experiments is shown. (**D**) Bar graphs represent the percentages of DCF fluorescence intensities as determined using flow cytometry from three independent experiments. * *p* < 0.05 and ** *p* < 0.01 indicate a significant difference between palmitate and non-palmitate-treated cells. ^##^ *p* < 0.01 indicates a significant difference between palmitate/G-Rc-treated cells and cells treated with palmitate only. Representative immunoblots (**E**) and bar graph quantitative assessment (**F**) of major signal transduction molecules involved in the MAPK (pJNK1/2^T183/Y185^, JNK1/2, pp38^T180/Y182^, p38) and NF-κB (pIKK^S176/S180^, IKK, pNF-κB p65^S536^, NF-κB p65) signaling pathways and β-Actin as a loading control in fully differentiated UB/OC-2 treated with palmitate alone, G-Rc alone, or palmitate in combination with G-Rc. Data is representative of at least three independent experiments. ** *p* < 0.01 indicates a significant difference between palmitate and non-palmitate-treated cells. ^##^ *p* < 0.01 indicates a significant difference between palmitate/G-Rc-treated cells versus cells treated with palmitate only.

Inflammatory processes, oxidative stress, and apoptosis are among the most common causes of cochlear hair cell death and the associated hearing loss. Given the well-established antioxidant and anti-inflammatory properties of ginsenosides, we examined changes in cell survival and proliferation in response to treatment with palmitate and G-Rc separately or in combination (Figure 4A,B). Palmitate induces inflammatory and cell death pathways in a plethora of cells via several mechanisms [30,31,32,33]. In this study, we demonstrated that treatment of cells with the commonly used ROS scavenger N-acetyl-L-cysteine (NAC) [34] alleviated palmitate-induced ROS production and inflammation (Figure S1A–C), yet failed to prevent palmitate-induced endoplasmic reticulum (ER) stress and apoptosis. Similarly, inhibition of ER stress with the chemical chaperone 4-phenylbutyric acid (4-PBA) [35] did not prevent palmitate-induced ROS production nor inflammation and had a minimum effect on apoptosis as judged by the expression of cleaved caspase-3 (cCasp3) (Figure S1C). On the other hand, treatment of differentiated UB/OC-2 with a pan-caspase inhibitor (carbobenzoxy-valyl-alanyl-aspartyl-[O-methyl] -fluoromethylketone; Z-VAD-FMK) [36] prevented caspase-3 cleavage, with no changes in ROS levels, inflammation, and ER stress (Figure S1A–C). Taken together, our findings demonstrate that palmitate induces UB/OC-2 apoptotic cell death via several mechanisms. We also observed that palmitate treatment abolished cell survival and proliferation of differentiated (Figure 4A) and undifferentiated (Figure 4B) cochlear hair cells, respectively. Treatment with G-Rc, on the other hand, alleviated the effects of palmitate on cell survival and proliferation (Figure 4A,B). Additionally, we investigated the effects of G-Rc treatment on palmitate-induced oxidative stress and inflammation in differentiated UB/OC-2 cochlear hair cells. We first examined changes in ROS production in response to palmitate treatment alone and in combination with G-Rc using flow cytometry (Figure 4C,D). Endogenous ROS production was quantified using 2′,7′-dichloro-dihydrofluorescein diacetate (DCH_2_F-DA). In the presence of ROS, non-fluorescent DCH_2_F-DA gets converted into its fluorescent 2′,7′-dichlorofluorescein (DCF) form that can be quantified using fluorometry. Treatment of UB/OC-2 cells with palmitate resulted in increased ROS production as judged by the increase in DCF levels (Figure 4C,D). Consistent with these findings, palmitate increased the phosphorylation and activation of key signaling molecules in the inflammatory pathway, namely the inhibitor of NF-κB kinase (IKK) and NF-κB p65 as well as the mitogen-activated protein (MAP) kinases p38 and c-Jun N-terminal kinase (JNK1/2) (Figure 4E,F). On the other hand, cells treated with G-Rc and palmitate exhibited a significant reduction in ROS production (Figure 4C,D) and activation of IKK, NF-κB p65, and MAP kinases (Figure 4E,F), further validating the antioxidative and anti-inflammatory properties of G-Rc in [differentiated] cochlear hair cells. 

### 2.5. G-Rc Alleviates Palmitate-Induced ER Stress and Apoptotic Cell Death

**Figure 5:**
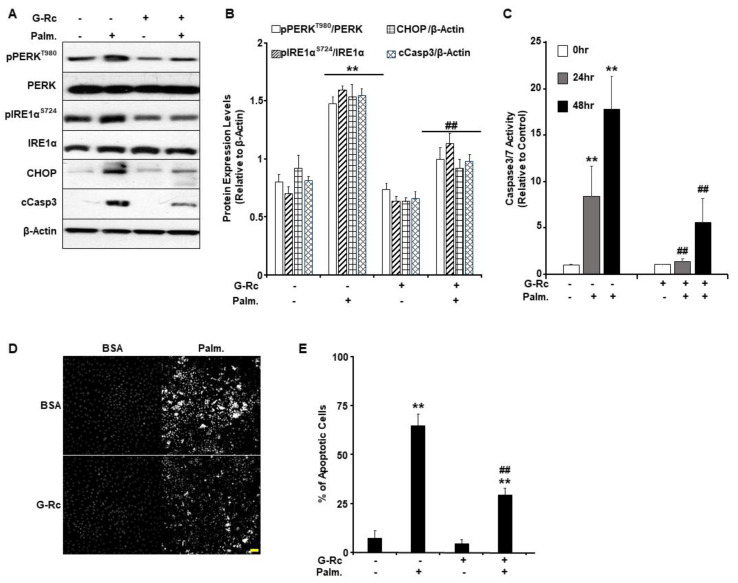
G-Rc alleviates palmitate-induced ER stress and apoptotic cell death. (**A**) Total cell lysates from fully differentiated UB/OC-2 cells treated with palmitate alone, G-Rc alone, palmitate in combination with G-Rc, or neither were immunoblotted for for markers of ER stress and apoptosis (pPERKT980, PERK, pIRE1αS724, IRE1α, CHOP, cCasp3) and β-Actin as a loading control. Representative immunoblots from three independent experiments are shown. (**B**) Bar graphs represent pPERK^T980^/PERK, pIRE1α^S724^/IRE1α, CHOP/β-Actin, and cCasp3/β-Actin as means + SEM. ** *p* < 0.01 indicates a significant difference between palmitate and non-palmitate-treated cells. ^##^ *p* < 0.01 indicates a significant difference between palmitate/ G-Rc-treated cells versus cells treated with palmitate only. (**C**) Casp3/Casp7 activity in fully differentiated UB/OC-2 treated with palmitate, G-Rc alone, palmitate in combination with G-Rc, or control cells. Data is representative of at least three independent experiments. ** *p* < 0.01 indicates a significant difference between palmitate and non-palmitate-treated cells. ^##^ *p* < 0.01 indicates a significant difference between palmitate/G-Rc-treated cells versus cells treated with palmitate only. (**D**,**E**) Representative images (**D**) and quantification (**E**) of chromatin condensation in Hoechst-stained, fully differentiated UB/OC-2 cells treated with palmitate alone, G-Rc alone, or palmitate in combination with G-Rc, or control cells. Scale bar: 50 μm. Images are representative of at least three independent experiments. ** *p* < 0.01 indicates a significant difference between palmitate and non-palmitate-treated cells. ^##^ *p* < 0.01 indicates a significant difference between palmitate/G-Rc-treated cells versus cells treated with palmitate only.

ER stress in the injured cochlea is suggested to promote the escalation from inflammation to cell death [37]. It has been reported that pharmacological inhibition of ER stress alleviated cochlear hair cell death and hearing loss in an experimental model of erlong (erl) mutant mice [38,39]. Recently, palmitate was demonstrated to elicit the unfolded protein response (UPR)- and ER stress-induced cell death in various cell types [40]. Therefore, we sought to examine the effect of G-Rc treatment on the activation of ER stress in response to palmitate treatment in differentiated UB/OC-2 cells. In line with published reports [41,42], palmitate induced the activation of ER stress in control cells, as judged by increased phosphorylation of ER transmembrane proteins, particularly protein kinase RNA-like endoplasmic reticulum kinase (PERK) and inositol-requiring enzyme 1α (IRE1α), and the upregulation of C/EBP homologous protein (CHOP) (Figure 5A,B). Notably, G-Rc treatment mitigated palmitate-induced ER stress as assessed by reduced phosphorylation of PERK and IRE1α as well as a decrease in CHOP and cCasp3 expression (Figure 5A,B). Together, these findings demonstrate a reduction in palmitate-induced ER stress in G-Rc-treated UB/OC-2 cells.

Since it has been shown that ER stress can regulate apoptosis in various biological systems, we sought to examine the effects of G-Rc on palmitate-induced cell death [43]. We used the physiologically relevant dose of G-Rc (25 µg/L) to examine changes in the activity of apoptotic effector caspases (Casp) 3 and 7 in differentiated UB/OC-2 cells upon treatment with palmitate (0.5 mM) for 24 and 48 h. Caspase3/7 activity was significantly elevated in response to palmitate treatment after 24 and 48 h compared to non-treated control cells (Figure 5C). On the other hand, differentiated UB/OC-2 cells treated with both G-Rc and palmitate showed a significant reduction in Casp3/Casp7 activity at 24 and 48 h compared to cells treated with palmitate alone (Figure 5C). These findings suggest that G-Rc can reduce palmitate-induced apoptosis by attenuating caspase activity.

It is well-established that the activation of effector caspases leads to chromatin condensation and DNA fragmentation [44]. Thus, we used Hoechst stain and fluorescence microscopy to examine changes in chromatin condensation in differentiated UB/OC-2 cells upon treatment with palmitate (0.5 mM). Greater fluorescence denotes increased levels of chromatin condensation and, therefore, a greater percentage of apoptotic cells in response to palmitate treatment. The percent of apoptotic cells was significantly higher in palmitate-treated cells compared to non-palmitate-treated cells (Figure 5D,E). On the other hand, cells co-treated with G-Rc and palmitate exhibited a significant reduction in the percentage of apoptotic cells compared to cells treated with palmitate alone (Figure 5D,E). Collectively, our findings demonstrate that G-Rc treatment ameliorates palmitate-induced oxidative stress, inflammation, ER stress, and apoptosis in differentiated cochlear hair cells.

## 3. Discussion

Hearing loss affects 30 million Americans with an estimated two-thirds of individuals over the age of 70 experiencing some extent of hearing loss [1,2]. Hearing loss is correlated with different pathologies that are associated with chronic inflammation such as CVD, T2D, and Alzheimer’s disease [6]. SNHL is largely attributed to an increase in apoptotic cell death due to injury or other pathophysiological disturbance to hair cells and cochlear function. Cochlear hair cells, located within the organ of Corti, are responsible for transducing fluid sound waves into neural electric signals that are conducted through afferent nerve fibers to provide sensory input to the spiral ganglion. Cochlear cell dysfunction is attributed to multiple causes, such as intense noise, aging, ototoxic drugs, and perhaps chronic inflammation. It often manifests in the inner ear as increased oxidative damage, enhanced inflammation, and reduced blood circulation, which exacerbates the damage. Protecting cochlear hair cells from damage may prevent or alleviate the effects of SNHL. 

Much of the literature surrounding the treatment of SNHL refers to the use of systemic and intratympanic steroid treatments, which are aimed at treating sudden sensorineural hearing loss, not acquired SNHL [45,46]. Most treatment approaches model the same strategy of blocking inflammation employed for the protection of existing cochlear hair cells, which is the aim of the current study. Due to the inability of cochlear hair cells to regenerate after death, combatting the loss of cochlear hair cells is the primary target for the prevention of SNHL and its progression [47]. Research into the mechanisms of mammalian cochlear hair cell repair and regeneration for treating SNHL is ongoing but has not yielded human-applicable therapies [48,49]. Treatment options for patients that currently experience moderate-to-severe hearing loss include the use of hearing aids while cochlear implants are recommended for patients with severe-to-profound hearing loss. Even though these options provide necessary improvements to the hearing threshold and quality of life in patients with SNHL, only an estimated 15% and 5% of eligible patients utilize hearing aids and cochlear implants in the U.S., respectively [1,50]. Such low utilization is attributable to negative social stigma, the difficulty of use, low referral rate to audiology professionals, and cost [1,50]. Therefore, the employment of novel therapies combined with existing strategies must be explored to overcome these boundaries and to provide effective treatment while also protecting vulnerable populations from SNHL.

A large body of evidence supports the use of phytochemicals as a potential therapy to combat hearing loss due to their anti-inflammatory and antioxidant properties. Indeed, there is extensive evidence for phytochemicals attenuating hearing loss across a wide range of compounds and models. Epicatechins and epigallocatechin gallate (EGCG), from *Camellia sinensis,* are shown to reduce hair cell death, ROS generation, and expression of p53, NF-κB, TNF-α, cyclooxygenase-2 (COX-2), and Casp3 in cochlear UB/OC-1 and HEI-OC1 cells as well as in Sprague-Dawley rats and zebrafish [3]. Curcumin was also able to reduce apoptosis in HEI-OC1 cells, which was attributed to its ability to reduce ROS production [51]. Similarly, the use of *Ginkgo biloba* led to beneficial protective effects both in vitro and in vivo. Comparable to other phytochemicals, quercetin acts on the apoptosis pathway by reducing the activity of Caspases 3, 8, and 9 and is shown to increase blood flow to the cochlea [52,53]. Sufficient blood circulation to the inner ear is important for decreasing the incidence of cochlear insult while also playing a role in the clearance of inflammatory molecules. Along with increased blood flow, quercetin has been shown to inhibit markers of inflammation such as IL-1β, IL-6, TNF-α, and COX-2 [3]. Likewise, rosmarinic acid and tanshinone IIA from *Salvia miltiorrhiza* also demonstrate the profound impact of phytochemicals on cochlear hair cell survival in vitro and in vivo by reducing caspase activity and auditory brainstem response (ABR) hearing thresholds [3]. Additionally, treatment of HEI-OC1 cells with tanshinone IIA led to a reduction in ROS generation, inhibition of NF-κB p65 nuclear translocation, and p53/p21 activation [54]. Taken together, these findings provide evidence supporting the therapeutic potential of phytochemicals for reducing and preventing damage to and loss of function of cochlear cells in response to pro-inflammatory and pro-apoptotic stimuli. 

*P. ginseng* and its bioactive molecules, ginsenosides, are shown to be anti-inflammatory and antioxidants. Our data demonstrate that UB/OC-2 primary murine sensory hair cells are a viable model for investigating the effects of G-Rc on hearing loss due to their ability to differentiate into cochlear hair cells. Our findings also establish that, at physiologically relevant doses, G-Rc promotes cell differentiation and preserves cell survival and cell cycle progression under palmitate-induced pro-inflammatory conditions. In addition, G-Rc treatment reduced ROS generation and alleviated palmitate-induced expression of markers of inflammation and ER stress. The activity of Casp3 in response to palmitate was also attenuated in cells treated with G-Rc, thus supporting the previously reported anti-apoptotic property of G-Rc [55]. Due to a lack of data on G-Rc specifically, we will here assess the evidence supporting the implication of using isolated ginsenosides or ginseng as a whole plant-based food for the prevention and treatment of hearing loss. Notably, our findings are in line with previous reports showing that treatment with other ginsenosides reduces ROS, Casp3 activity, NF-κB activation, and apoptosis in cochlear hair cells. Indeed, related studies conducted on cisplatin and gentamicin-induced injury of HEI-OC1 cells revealed that KRG protected cells against cisplatin-induced ROS generation [56] and the induction of apoptotic cell death. Likewise, a study by Kim and colleagues demonstrated that KRG protected HEI-OC1 cells against cisplatin-induced ROS generation and Casp3 activation [57]. In another study, Choung and colleagues showed that two ginsenosides found in *P. ginseng*, G-Rb1 and Rb2, were effective at preventing gentamicin-induced apoptosis in HEI-OC1 cells in a dose-dependent manner as determined by a reduction in the cleavage of poly (ADP-ribose) polymerase (PARP) [58]. Collectively, these findings reveal a novel role for ginseng in protecting cochlear cells against oxidative stress damage and cell death and warrant additional investigations into ginseng’s role in cochlear cell function and hearing loss in response to injury.

Our study corroborates the overall anti-inflammatory and anti-apoptotic properties of ginseng. While the exact molecular mechanisms are yet to be determined, previous reports have established that KRG treatment inhibits cisplatin-induced expression of pro-inflammatory cytokine IL-6 and attenuates NF-κB activation [57]. The same study also postulated that KRG may alleviate inflammation through the suppression of cytokine expression and IKK phosphorylation to inhibit the subsequent translocation of NF-κB to the nucleus, where it acts as a transcription factor to upregulate inflammatory and apoptotic genes [57]. 

While few studies examine the role of ginseng and its constituents in cochlear cell function, the majority of evidence on ginseng’s link to inflammation originates from its role in alleviating and preventing Parkinson’s disease (PD) -related inflammation of the brain and colon. KRG was shown to suppress pro-inflammatory cytokines IL-1β and TNF-α in mouse models of PD-related colitis [59]. A follow-up study by the same group showed that treatment with KRG not only relieved colitis but, in doing so, alleviated inflammation in the substantia nigra brain region in a PD mouse model [60]. KRG was also shown to reduce inflammation by inhibiting NF-κB activation in a rat model of rotenone-induced PD, leading to an increase in locomotor activity [61]. Furthermore, ginseng has been implicated in relieving stress-induced neuroinflammation by attenuating pro-inflammatory signaling in heat-stressed rats and decreasing COX-2 in the amygdala of a chronic restraint stress (CRS) rat model [62,63]. The CRS model also proposed a novel role of ginsenosides in alleviating depression through their ability to inhibit the release of cytokines, chemokines, and other pro-inflammatory molecules in the microglia [63]. For instance, ginsenoside Rg3 (G-Rg3) was demonstrated to be effective in reducing the levels of TNF-α, IL-1β, and IL-6 and subsequent neutrophil accumulation in the bronchial tissue in a lipopolysaccharide (LPS)-induced lung damage mice model [64]. Likewise, using a mice model of inflammatory bowel disease, Seong and colleagues demonstrated that fermented wild ginseng reduces the expression of pro-inflammatory cytokine at the mRNA and protein levels and suppresses macrophage infiltration through inhibition of NF-κB in response to LPS [65]. Kang and colleagues proposed that the anti-inflammatory properties of ginsenosides could result from their ability to promote the polarization of M2 macrophages while also inhibiting M1 polarization, leading to a quick resolution of inflammation [66]. Although the exact molecular mechanisms are yet to be determined, it has been proposed that G-Rg3 prevents LPS-induced loss of arginase-1 and COX-2 activation [66], which are both key enzymes in the process of M2 differentiation and polarization [67,68]. 

Our data are also in line with the reported anti-apoptotic properties of ginseng that seem to be widely attributed to increased expression of B-cell lymphoma-2 (Bcl-2) and the inhibition of caspase activity and other pro-apoptotic signals [69,70,71]. For instance, in a study by Luo and Luo looking at the effects of American ginseng on pancreatic β-cell function, IL-1β-induced β-cell death was significantly reduced by ginseng. These effects were concomitant with reduced Casp9 activation, increased Bcl-2 and ATP levels, and enhanced insulin production and secretion [69]. Likewise, red ginseng was shown to suppress apoptosis in the brain of immobilization-stressed mice by reducing the expression of the oxidative stress marker peptidyl arginine deiminase type IV (PADI4) and inhibiting the activation of pro-apoptotic proteins, namely p53, Casp3, and JNK [70]. Although several pathways have been proposed as potential molecular mechanisms that mediate the anti-apoptotic effects of ginsenosides, they all seem to be centered toward the reduction of oxidative stress and the activation of antioxidant response and defense mechanisms. This includes the stimulation of glutathione, catalase, glutathione S-transferase, and glutathione peroxidase [71]. Importantly, ginsenosides not only protect against apoptosis but also promote cell cycle progression and the activation of autophagy [71], a signaling pathway with a key role in the development and differentiation of cochlear cells, as well as the protection against oxidative stress and maintenance of homeostasis [72,73,74,75]. 

The upregulation of inflammatory pathways in cochlear hair cells has been proposed as a significant contributor to hearing loss [76,77]. Nevertheless, another potential mechanism of hearing loss discussed in the recent literature is the impairment of ER homeostasis [78]. The function of the ER can be impacted by a wide variety of stressors, such as increased synthesis of unfolded proteins, oxidative stress, and hypoxia, causing ER stress that triggers the UPR as an adaptive mechanism to maintain and restore the folding capacity of the ER [79,80]. The UPR is mediated through three ER transmembrane proteins: PERK, IRE1α, and activating transcription factor 6 (ATF6) [81]. In the absence of stress, these proteins are maintained in an inactive state by binding their luminal domains to ER chaperone 78-kDa glucose-regulated protein 78 (GRP78) or immunoglobulin protein (BiP). However, under stress, these sensors are rendered active through their release from BiP, leading to a cascade of downstream signaling pathways [79]. Activated PERK phosphorylates eIF2α, which, in turn, inhibits mRNA translation and thus protein synthesis [82]. The translocation of ATF6 to the Golgi results in its cleavage, producing an active transcription factor that induces the expression of target genes involved in the degradation of unfolded proteins. Meanwhile, the activation of IRE1α leads to cleavage of X-box binding protein 1 (XBP1) mRNA, forming an active transcription factor that induces the expression of ER chaperones [79]. The combined action of these mechanisms reduce ER stress and promote cell adaptation and survival. However, if the cells fail to restore ER homeostasis, the UPR induces apoptotic signaling pathways [79,80]. Chronic ER stress has been implicated in several pathological conditions, including hearing loss [78,83]. In a mouse model of age-related hearing loss, morphological alterations in the cochlea were concomitant with an increase in ER stress markers and the ER stress-related apoptotic proteins, including Casp12 and the transcription factor CHOP [78]. Consistent with these findings, inhibition of ER stress in a rat model of cisplatin-induced ototoxicity using tauroursodeoxycholic acid (TUDCA) alleviated cisplatin-induced hearing loss [84]. TUDCA also attenuated ER stress-mediated apoptosis in cochlear explants and HEI-OC1 cells [85] and alleviated gentamicin-induced cell death in HEI-OC1 cells [86]. Likewise, the inhibition of ER stress using the murine mesencephalic astrocyte-derived neurotrophic factor (MANF) protected outer hair cells against cell death and prevented hearing loss [87]. Moreover, in a mouse model of hearing loss with a novel cadherin-23 (Cdh23) mutation erl mice treated with an intraperitoneal salubrinal injection exhibited a reduction in outer hair cell death and hearing loss [39]. Salubrinal has been shown to protect against ER stress-induced apoptosis and promote cell survival by attenuating translation through the phosphorylation of eIF2α, thus allowing proteostasis to be re-established and avoiding ER stress-induced apoptosis [39]. 

The inhibition of ER stress-induced apoptosis is critical for preventing hearing loss. Here we demonstrate that G-Rc inhibits palmitate-induced ER stress, concomitant with a reduction in apoptosis. Our findings align with previous reports demonstrating the potential of various ginsenosides to attenuate ER stress. Chen and colleagues demonstrated that the attenuation of ER stress by G-Rb1 and its derivative CK results from the inhibition of ROS [88] production in adipose tissue exposed to high glucose levels. The reduction in ROS production was accompanied by a decrease in IRE1α and PERK activation as well as a protection against the activation of the NLR family pyrin domain-containing protein 3 (NLRP3) inflammasome [88]. Likewise, treatment of cardiac myocytes with *Panax notoginseng* saponins (PNS) alleviated thapsigargin-induced ER stress and enhanced autophagy [89]. These protective effects were associated with a reduction in ROS accumulation and the prevention of alterations to Ca^2+^ homeostasis [89]. In addition, treatment with the ginsenoside Mc1 attenuated ER stress in the liver of obese mice, as demonstrated by a reduction in GRP78, CHOP, and Bcl-2-associated X protein (Bax)/Bcl ratio [90]. Similarly, G-Rb1 and CK both enhanced autophagy and prevented ROS production and inflammation in an ER stress-dependent mechanism [91]. Taken together, these studies suggest the attenuation of ER stress as a potential mechanism enabling ginsenosides to protect against stress-induced cell death and loss of function leading to hearing impairment. Further research is required to validate our findings in animal models and clinical trials. 

## 4. Materials and Methods

### 4.1. Reagents

Unless indicated otherwise, we obtained most chemicals from Millipore-Sigma (Burlington, MA, USA). Dulbecco’s Modified Eagle Medium (DMEM), penicillin-streptomycin, fetal bovine serum (FBS), and trypsin were all obtained from Invitrogen (Carlsbad, CA, USA). Primary and secondary antibodies with their dilutions, hosts, and sources are summarized in Table 1. Forward and reverse primers used for qRT-PCR are listed in Table 2 and were purchased from Fisher Scientific (Hampton, NH, USA).

### 4.2. Cell Culture

UB/OC-2 murine cells were derived from cochlear sensory epithelium by Dr. Matthew C Holley (University of Bristol and University of Sheffield [27,28]) and purchased from XimBio (London, UK). Cells were maintained at 33 °C in a humidified atmosphere of 10% CO_2_ in DMEM supplemented with 10% FBS, Glutamax (2 mM), and sodium pyruvate (1 mM). Differentiation of cochlear hair cells was induced as previously described with modification [27,92]. Briefly, cells were transferred to 38 °C and cultured for an additional period of 15 days. By then, cells exhibited a fully differentiated phenotype as judged by the expression of cochlear hair cell markers, namely, Vimentin, Myosin VIIa, Hsc70, Espin, Annexin IV, and Sox2. Cell culture media was replaced every 48 h, and all experiments were conducted at least three times on cells between passages 3 and 8. When mentioned, cells were pre-treated with G-Rc alone for 12 h, then exposed to the specified concentration of palmitate complexed with fatty acid-free bovine serum albumin (BSA FAF; Fisher Scientific) for the indicated duration [93]. G-Rc was dissolved in cell culture media at a concentration of 25 mg/mL by ultrasonication. In all experiments involving treatments with palmitate, control cells were treated with similar volumes of BSA FAF as vehicle control.

### 4.3. Protein Extraction and Immunoblots

Cells were lysed in radioimmunoprecipitation assay (RIPA) buffer enriched with freshly prepared solutions of proteases and phosphatase inhibitors (RPI Corp., Mount Prospect, IL, USA), PMSF (1 mM), and NaF (15 mM). Next, samples were sonicated twice (10 s each) on ice and cleared by 10 min centrifugation at 15,000× *g* at 4 °C. Then, the protein concentration was determined using the bicinchoninic acid assay kit (Pierce Chemical, Dallas, TX, USA). A total of 10 μg of proteins were resolved in electrophoresis and then transferred to PVDF membranes. Membranes were blocked with TBS-T containing 5% BSA and 0.1% Tween, pH: 7.4, for 45–60 min at room temperature (RT) before incubation with the indicated primary antibodies (Table 1) for 1 h at RT. After three wash cycles with TBS-T (5 min each), membranes were incubated with the appropriate secondary antibodies for 1 h at RT, then washed 4 times (15 min each) with TBS-T. Luminata™ Western Chemiluminescent HRP Substrate (Millipore Corp., Billerica, MA, USA) was used to visualize proteins, and band intensity was quantified using Fluorchem software (Alpha Innotech Corp., San Jose, CA, USA). 

### 4.4. RNA Isolation and qRT-PCR

mRNA was extracted from cells using TRIzol reagent (Invitrogen), pelleted in RNase-free water following the manufacturer’s instructions, and quantified using NanoDrop^®^ ND1000 spectrophotometer (Thermo Fisher Scientific Inc., Piscataway, NJ, USA). Five µg of RNA was used for cDNA synthesis using iScript™ cDNA Synthesis Kit (BioRad; Hercules, CA, USA). All genes’ expressions were performed by qRT-PCR using the SsoAdvanced™ Universal SYBR^®^ Green Supermix (BioRad) and BioRad CFX96™ system, as previously described [94]. We evaluated the relative abundance of mRNA of the indicated genes using respective primers (Table 2) via the 2^−ΔΔCt^-method. For each gene, the expression of their corresponding mRNA was normalized to *tata*-*box binding protein* (*Tbp*) as previously described [95].

### 4.5. Assessment of Oxidative Stress

The generation of ROS was measured using DCH_2_F-DA (10 μM), which produces green fluorescence when oxidized by ROS [96]. Immunofluorescence levels for 5000 cells were collected using Guava^®^ easyCyte Flow Cytometer (Millipore-Sigma) on the PM1 channel and quantified using the InCyte™ and GuavaSuite Software package (Luminex Corp.; Austin, TX, USA). Only data from viable cells (negative for 7-AAD staining) was used.

### 4.6. Cell Proliferation Assay

Cell proliferation assays were performed using the sulforhodamine B (SRB; Millipore-Sigma) method as previously described with modification [97]. Briefly, an equal number of undifferentiated UB/OC-2 cells (1 × 10^6^) were seeded in 6 well plates. Six hours later, cells were treated with the indicated concentrations of palmitate alone or in combination with G-Rc and incubated at 38 °C (10% CO_2_) for the indicated time. Cells were fixed with 17% trichloroacetic acid in phosphate-buffered saline (PBS) and then washed twice with ice-cold PBS to stop the treatment. Intracellular proteins were stained for 10 min at room temperature using 0.4% SRB dissolved in 1% acetic acid. Excess SRB stain was removed by rinsing the plates thoroughly with running tap water. Plates were air-dried overnight before dissolving the stain in 10 mM Tris (pH 9.0). Cells that survived the stress were quantified by measuring the number of intracellular proteins using the Synergy™ HTX Multi-Mode microplate reader (BioTek Instruments, Inc., Winooski, VT, USA) at a wavelength of 540 nm. The relative survival rates of cells were determined by dividing the absorbance observed for a given treatment by the absorbance detected in control cells and expressed as a fold change.

### 4.7. Cytotoxicity Assay

The MTT (3-[4,5-dimethylthiazol-2-yl]-2,5-diphenyltetrazolium bromide) cytotoxicity assay was performed as previously described with modification [98]. Briefly, 1 × 10^4^ cells were plated in a 96-well plate for 24 h. Then, a freshly prepared solution of palmitate alone (0.5 mM) or in combination with G-Rc was added for the indicated time. The experiment was terminated by adding 40 μL of the MTT solution (5 mg/mL) to each well and incubating for 4 h. Next, the cell culture medium was discarded, and the dye was dissolved in 100 μL of sodium dodecyl sulfate solution (10%) overnight at 37 °C. Relative cytotoxicity was determined by measuring the absorbance at 570 nm using the Synergy™ HTX Multi-Mode microplate reader and expressed as a fold change.

### 4.8. Morphological Analysis of Apoptosis

When indicated, UB/OC-2 cells were pre-treated with G-Rc for 12 h and then exposed to a freshly prepared solution of palmitate alone (0.5 mM) or in combination with G-Rc for the indicated duration. Treatments were stopped by washing the cells with PBS and cells were labeled with Hoechst 33258 (50 µg/mL in PBS) (blue-green fluorescence). Hoechst binds to condensed nuclear chromatin and was used to visualize apoptotic cells (green fluorescence) by fluorescence microscopy (Leica DMI8, Leica Microsystems Inc., Buffalo Grove, IL, USA) [99]. For each condition, at least 300 cells were counted. Percentages of apoptotic cells were calculated relative to total cells.

### 4.9. Cell Cycle Analysis

Cell cycle analysis was conducted by assessing the DNA content of cells stained with propidium iodide (PI) as previously described with modification [100]. Briefly, UB/OC-2 cells were exposed to a freshly prepared solution of palmitate alone (0.5 mM) or in combination with G-Rc for the indicated duration, then washed with PBS and fixed overnight in 70% ethanol at 4 °C. Next, cells were washed twice with ice-cold PBS and incubated in a freshly prepared RNase solution (10 mM Tris-HCl, pH 7.4 containing 100 U/mL of DNase-free RNase A (Applied Biosystems, Austin, TX, USA)) for 30 min at 37 °C. Cells were then washed twice with ice-cold PBS and incubated in a solution of PI (10 μg/mL in PBS) overnight at 4 °C protected from light. DNA content was measured by assessing the fluorescence intensity of PI using the Guava^®^ easyCyte flow cytometer on PM2 channel. DNA histogram analysis was performed on 5000–10,000 cells using the InCyte™ and GuavaSuite Software package, and the proportions of cells with one or two copies of their chromosomal DNA were calculated.

### 4.10. Statistical Analyses

Statistical analyses were conducted using JMP data analysis software (SAS Institute Inc., Cary, NC, USA), and an unpaired heteroscedastic two-tail Student’s *t*-test was conducted for a two-group comparison, while ANOVA (with post-hoc analysis) was conducted for a multi-group comparison. Data are shown as means + standard error of the mean (SEM). The level of significance was set at *p* < 0.05; the single symbol (*) refers to *p* < 0.05 and double symbols (**) refer to *p* < 0.01.

## 5. Conclusions

Findings from the study validate the beneficial effects of ginsenosides in protecting cochlear hair cells against oxidative stress, ER stress, and apoptotic cell death and warrant additional investigations into the mediating molecular mechanisms. 

## Figures and Tables

**Table 1:** List of antibodies used in the reported experiments.

Antibody	Source	Catalog Number	Observed MW (kDa)	Host	Dilution
Annexin IV	Santa Cruz Biotechnology	sc-46693	35	Mouse	1:500
CHOP	Santa Cruz Biotechnology	sc-7351	31	Mouse	1:5000
Cleaved Caspase-3	Cell Signaling Technology	9662	17	Rabbit	1:5000
Espin	Santa Cruz Biotechnology	sc-393469	27	Mouse	1:500
Hsc70	Santa Cruz Biotechnology	sc-7298	70	Mouse	1:1000
IKKα	Cell Signaling Technology	2682	87	Rabbit	1:1000
IRE1α	Cell Signaling Technology	3294	115	Rabbit	1:1000
IκBα	Cell Signaling Technology	4814	40	Mouse	1:1000
JNK1/2	Santa Cruz Biotechnology	sc-7345	46/54	Mouse	1:1000
Myosin VIIa	Santa Cruz Biotechnology	sc-74516	200	Mouse	1:1000
NF-κBp65	Cell Signaling Technology	8242	65	Rabbit	1:1000
p38	Santa Cruz Biotechnology	sc-7972	42	Mouse	1:1000
PERK	Cell Signaling Technology	3192	140	Rabbit	1:1000
Phosoho-IκBα^S32^	Cell Signaling Technology	2852	40	Rabbit	1:1000
Phospho-IRE1α^S724^	Abcam	ab 48187	115	Rabbit	1:10,000
Phospho-JNK1/2^T183/Y185^	Santa Cruz Biotechnology	sc-6254	46/54	Mouse	1:1000
Phospho-P38^T180/Y182^	Cell Signaling Technology	4511	43	Mouse	1:10,000
Phospho-PERK^T980^	Santa Cruz Biotechnology	sc-32577	160	Rabbit	1:1000
Phospho-PKM2^S37^	ThermoFisher	PA5-37684	61	Rabbit	1:500
Phospho-IKKα^S176/S180^	Cell Signaling Technology	2697	87	Rabbit	1:1000
Phospho-NF-κBp65^S536^	Cell Signaling Technology	3033	65	Rabbit	1:1000
Sox2	Santa Cruz Biotechnology	Sc-365823	35	Mouse	1:500
Vimentin	Santa Cruz Biotechnology	Sc-6260	58	Mouse	1:500
β-Actin	Santa Cruz Biotechnology	sc-47778	44	Mouse	1:20,000

**Table 2:** List of primers used in qRT-PCR experiments.

Gene	Forward 5′→3′	Reverse 5′→3′
*Hsc70*	GAAGGTGCTGGACAAGTGC	GCCAGCAGAGGCCTCTAATC
*Myo7a*	CTCAAGCTGCTCAGCAATCTATTT	GGAGCGCAAGTTTGTCATAAGT
*Tbp*	TTGGCTAGGTTTCTGCGGTC	GCCCTGAGCATAAGGTGGAA
*Vim*	CGGCTGCGAGAGAAATTGC	CCACTTTCCGTTCAAGGTCAAG

## Data Availability

All data are contained within the manuscript.

## Supplementary Materials

The supporting information can be downloaded at: https://www.mdpi.com/article/10.3390/ijms24087345/s1.

## Abbreviations

Sensorineural hearing loss (SNHL); cardiovascular disease (CVD); type 2 diabetes (T2D); interleukin-1β (IL-1β); interleukin-6 (IL-6); tumor necrosis factor-α (TNF-α); nuclear factor kappa B (NF-κB); traditional Chinese medicine (TCM); compound K (CK); ginsenoside Rb1 (G-Rb1); ginsenoside Rc (G-Rc); reactive oxygen species (ROS); TANK-binding kinase-1 (TBK1); interferon regulatory factor-3 (IRF-3); activating transcription factor-2 (ATF-2); Korean red ginseng (KRG); Purkinje cell protein 4 (PCP4); SRY-box 2 (Sox2); heat shock cognate 70 (Hsc70); quantitative real-time polymerase chain reaction (qRT-PCR); endoplasmic reticulum (ER); 2′,7′-dichlorodihydrofluorescein diacetate (DCH_2_F-DA); 2′,7′-dichlorofluorescein (DCF); inhibitor of NF-κB kinase (IKK); mitogen-activated protein (MAP); c-Jun N-terminal kinase (JNK1/2); erlong (erl); unfolded protein response (UPR); protein kinase RNA-like endoplasmic reticulum kinase (PERK); inositol-requiring enzyme 1α (IRE1α); C/EBP homologous protein (CHOP); cleaved caspase 3 (cCasp3); caspase (Casp); epigallocatechin gallate (EGCG); cyclooxygenase-2 (COX-2); auditory brainstem response (ABR); poly (ADP-ribose) polymerase (PARP); Parkinson’s disease (PD); chronic restraint stress (CRS); ginsenoside Rg3 (G-Rg3); lipopolysaccharide (LPS); B-cell lymphoma-2 (Bcl-2); peptidyl arginine deiminase type IV (PADI4); activating transcription factor 6 (ATF6); glucose-regulated protein 78 (GRP78); binding immunoglobulin protein (BiP); X-box binding protein 1 (XBP1); tauroursodeoxycholic acid (TUDCA); mesencephalic astrocyte-derived neurotrophic factor (MANF); cadherin-23 (Cdh23); NLR family pyrin domain containing protein 3 (NLRP3); panax notoginseng saponins (PNS); Bcl-2-associated X protein (Bax); Dulbecco’s Modified Eagle Medium (DMEM); fetal bovine serum (FBS); *tata*-*box binding protein* (*Tbp*); sulforhodamine B (SRB); phosphate buffered saline (PBS); propidium iodide (PI); standard error of the mean (SEM).
