# Supplementary material for: Ginsenoside Rc from Panax Ginseng Ameliorates Palmitate-Induced UB/OC-2 Cochlear Cell Injury
Source: Int J Mol Sci. 2023 Apr 16;24(8):7345. doi: 10.3390/ijms24087345 (PMC10139021; doi:10.3390/ijms24087345)
Supplement: Supplementary file 1 [file ijms-24-07345-s001.zip › ijms-2283969-supplementary.pdf]

Supplementary Materials: Figure S1

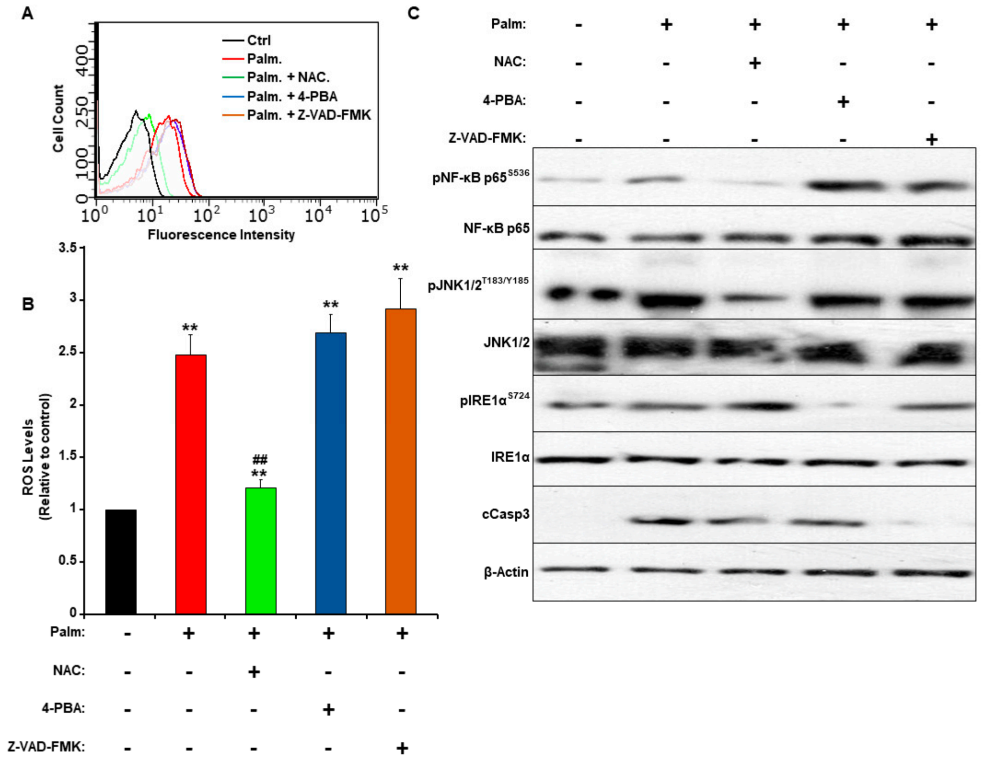

Figure S1. Palmitate induces cell death in UB/OC-2 cells via multiple mechanisms. **A-B)** Flow Cytometry assessment of ROS production as determined using CM- DCH2F-DA in fully differentiated UB/OC-2 cells treated with palmitate (0.5mM) or palmitate in combination with NAC (5mM), 4-PBA(0.5mM), or Z-VAD-FMK (10µM) for 24 hours. A representative histogram (**A**) for each treatment from three independent experiments is shown. Bar graphs (**B**) represent the percentages of DCF fluorescence intensities as determined using flow cytometry from three independent experiments. **p<0.01 indicate a significant difference between palmitate and non-palmitate-treated cells. ##p<0.01 indicate a significant difference between palmitate and NAC-treated cells and cells treated with palmitate only. **C**) Representative immunoblots of markers of inflammation (pNF-κBp65^{S536}, NF-κBp65; pJNK1/2$^{T183/Y185}$, JNK1/2,) , ER stress (pIRE1α^{S724}, IRE1α,), and apoptosis (cleaved Caspase 3), and β-Actin as a loading control in total cell lysates of fully differentiated UB/OC-2 cells treated with palmitate (0.5mM) or palmitate in combination with NAC (5mM), 4-PBA(0.5mM), or Z-VAD-FMK (10µM) for 24 hours. Representative immunoblots from at least three independent experiments are shown.
